# Supplementary material for: Multi-omics analysis reveals the interplay between intratumoral bacteria and glioma
Source: mSystems. 2024 Dec 11;10(1):e00457-24. doi: 10.1128/msystems.00457-24 (PMC11748541; doi:10.1128/msystems.00457-24)
Supplement: Supplemental Figures — Figures S1 to S10. [file msystems.00457-24-s0001.pdf]

Supplementary Figure

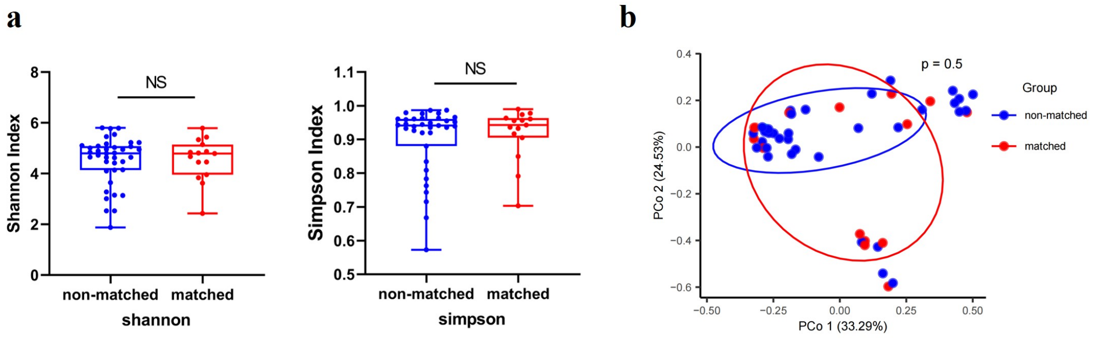

Figure S1 Microbial diversity between non-matched and matched groups in glioma tissue.

a α diversity differences were estimated by the Shannon, and Simpson indices. NS, not significant.

b PCoA was shown along the first two principal coordinates of Bray-Curtis distances. The P value was calculated by PERMANOVA. non-matched group (blue dots); matched group (red dots), where dots represent individual samples.

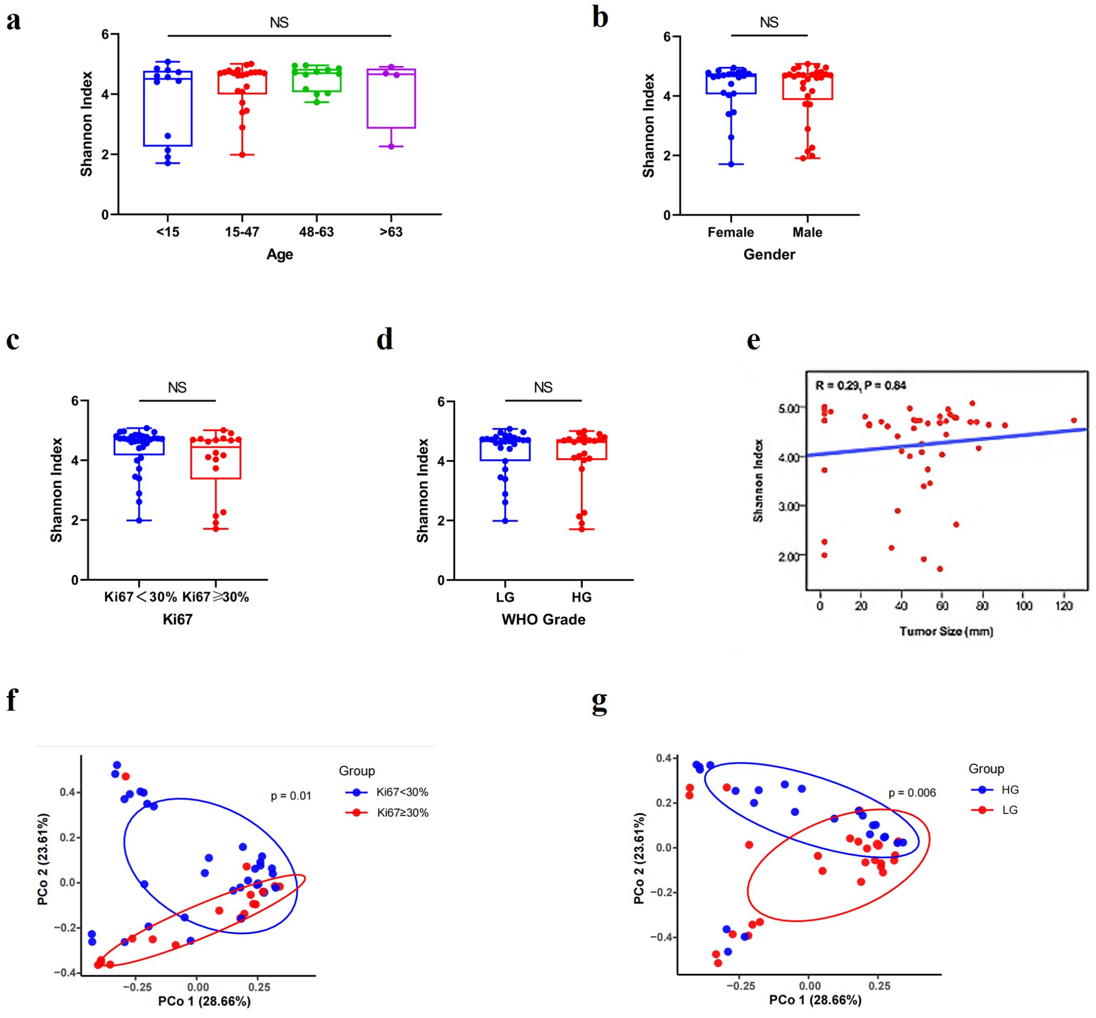

Figure S2 Association between intratumoral microbiota and clinical features of glioma**.**

a-e Alpha diversity boxplots (Shannon index) in glioma patients. Analysis of following factors: Age, Gender, Ki67, WHO grade, Tumor size.

f-g PCoA plots of intratumoral bacteria diversity in the Ki67<30% and Ki67≥30% glioma groups and in the low-grade and high-grade glioma groups. LG, WHO I - II; HG, WHO III – IV.

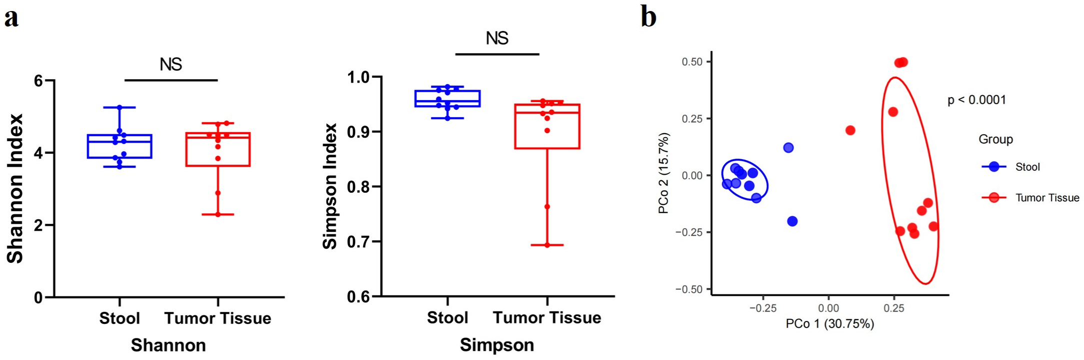

Figure S3 Microbial diversity between stool and tumor tissue samples in glioma.

a α diversity differences were estimated by the shannon, and simpson indices. $*P < 0.05$; NS, not significant.

b PCoA was shown along the first two principal coordinates of Bray-Curtis distances. The P value was calculated by PERMANOVA. Stool group (blue dots); Tumor Tissue group (red dots), where dots represent individual samples.

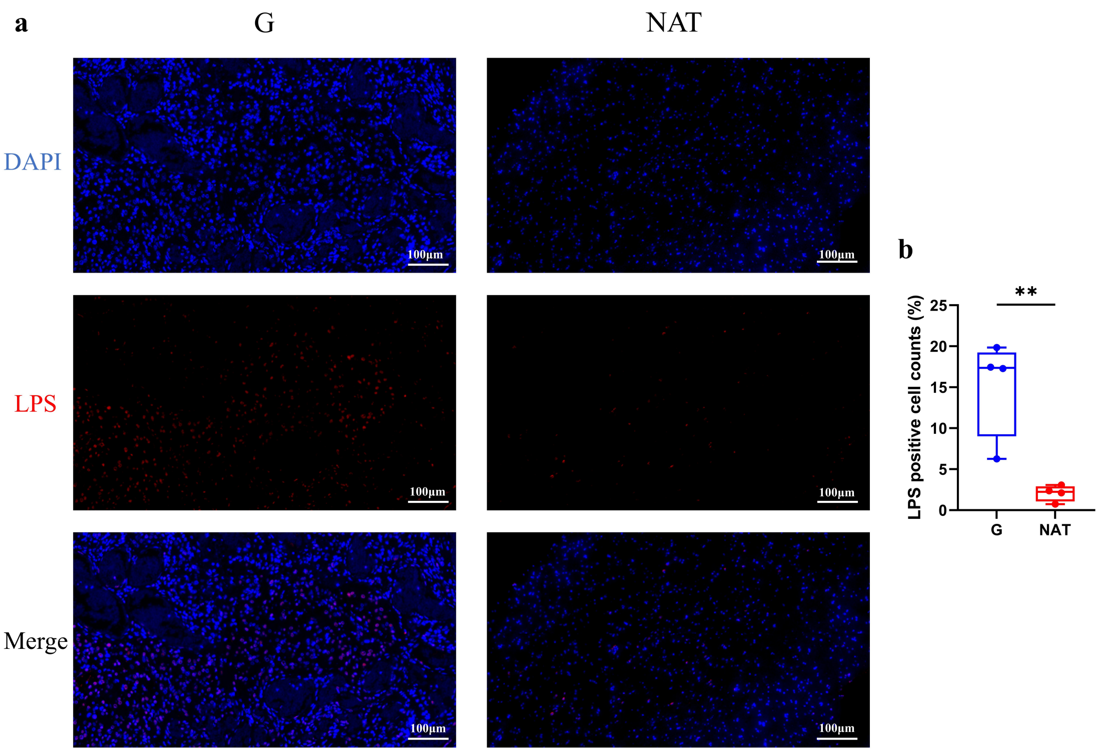

Figure S4 Immunofluorescence staining of LPS in human glioma tissue and adjacent normal brain tissue.

a Immunofluorescence staining of glioma tissue and adjacent normal brain tissue with anti-LPS antibody at 20X, respectively. LPS (red) and DAPI (blue). scale bar = 100 μm.

b Statistics results of cell counts of LPS positive cells after immunofluorescence staining experiments followed by panoramic scanning. Each symbol represents one sample. n=4; $^{**}P < 0.01$.

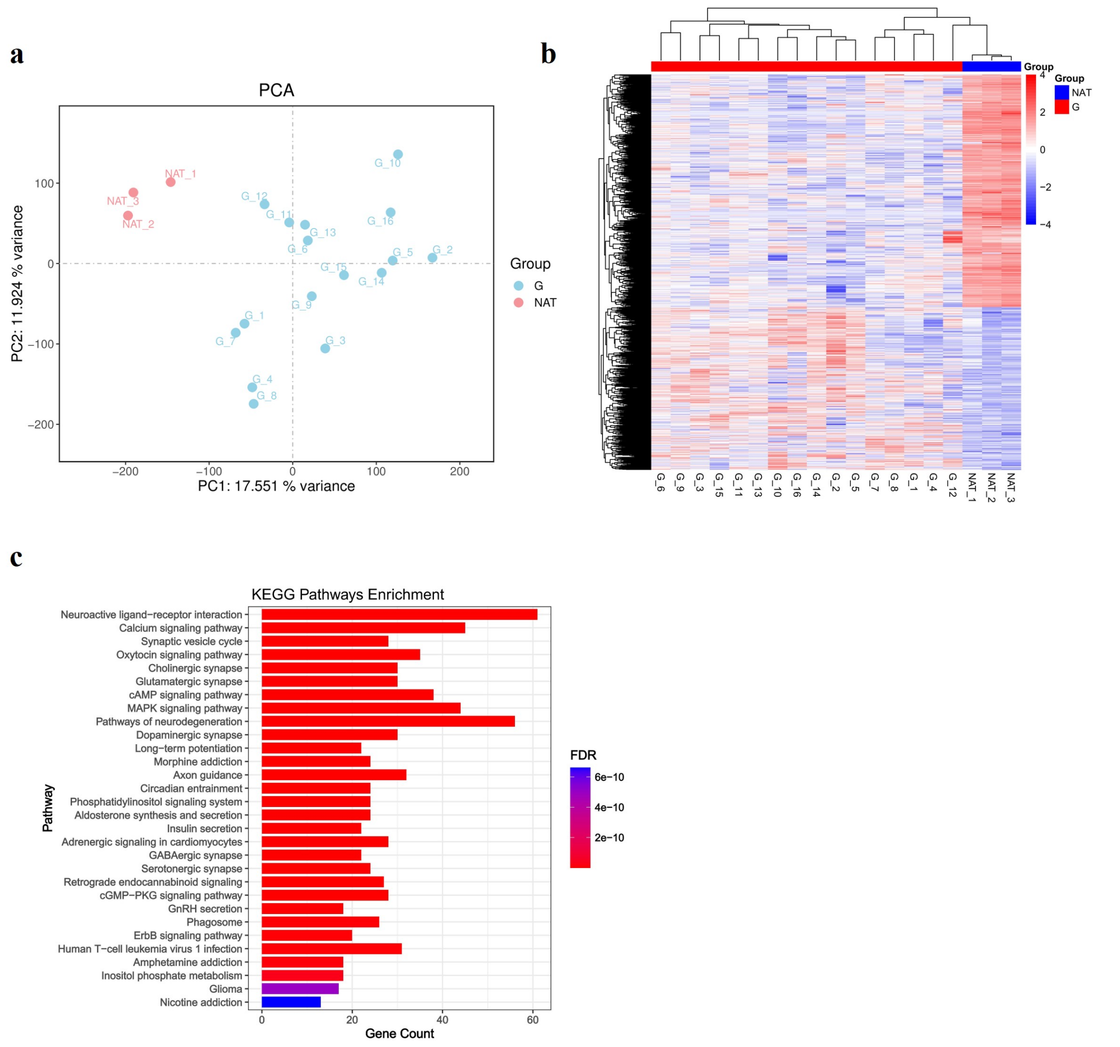

Figure S5 Profiling of host gene expression in human glioma tissue.

a PCA plot of transcriptomics data collected from human glioma tissue and adjacent normal brain tissue samples.

b Hierarchical clustering heatmap of differential expression genes between G and NAT group.

c Bar diagram of KEGG pathway enrichment analysis of all differential expression genes.

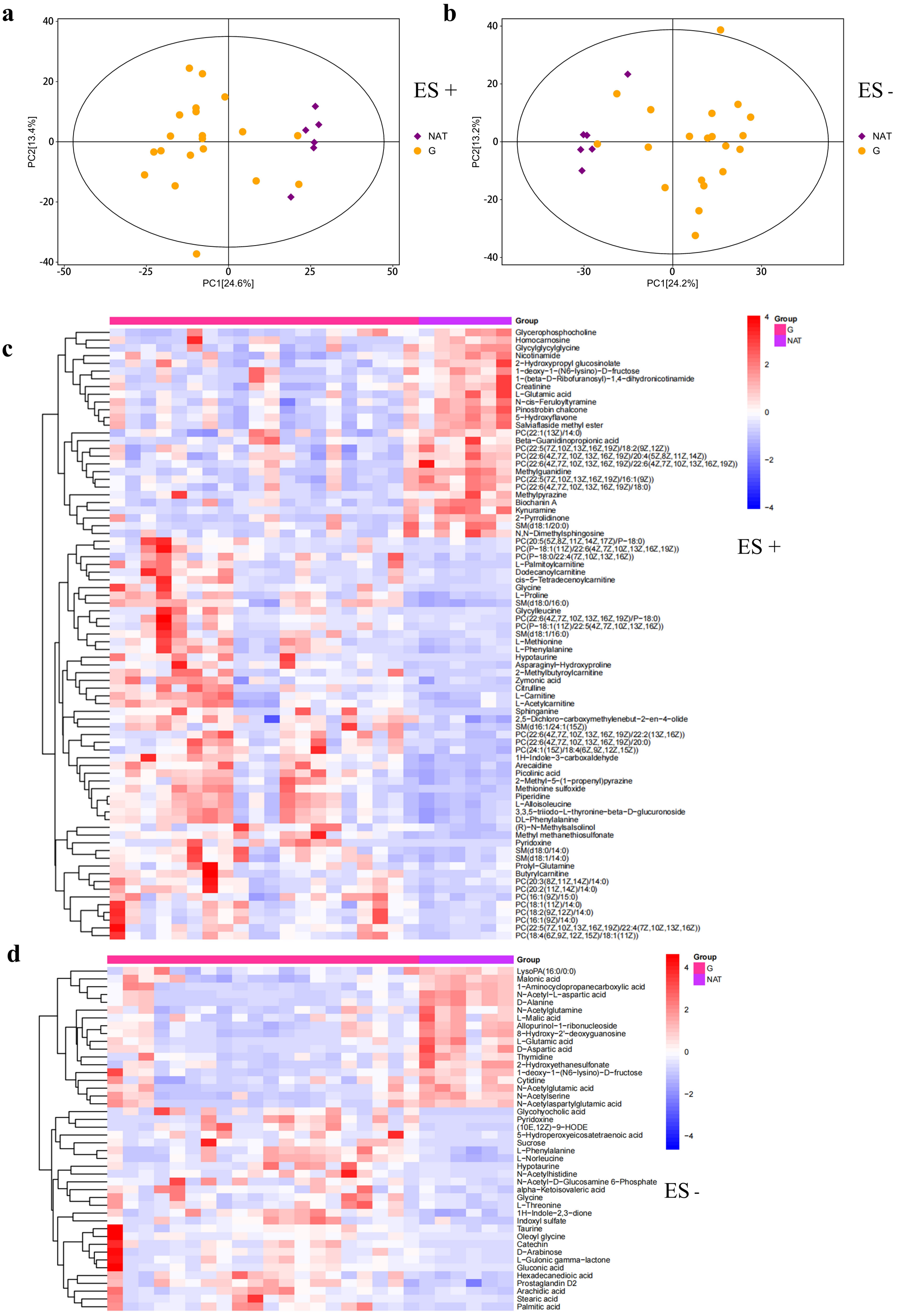

Figure S6 Metabolomic analysis of glioma tissue and adjacent normal brain tissue.

a-b Score scatter plot of PCA model between G and NAT group.

c-d Hierarchical clustering heatmap of differential metabolites between G and NAT group.

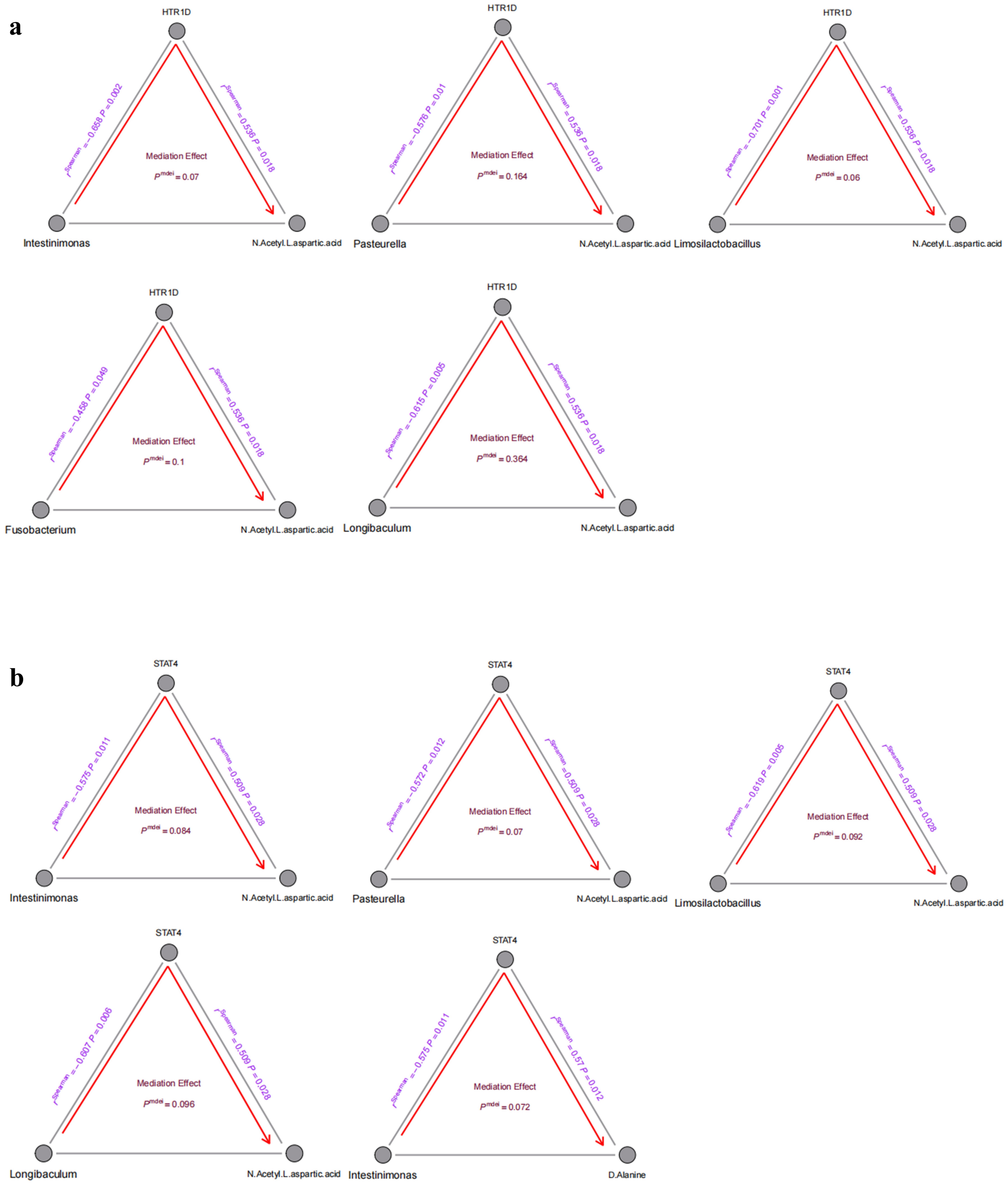

Figure S7 Diagram of Mediation analysis

a Pathways mediated through HTR1D.

b Pathways mediated through STAT4. The gray lines indicate the associations among bacteria, metabolites, and genes, with corresponding $r^{Spearman}$ values and *P*-values. The red arrowed lines indicate the bacterial effects on gene expression mediated by

metabolites, with the corresponding mediation *P*-values.

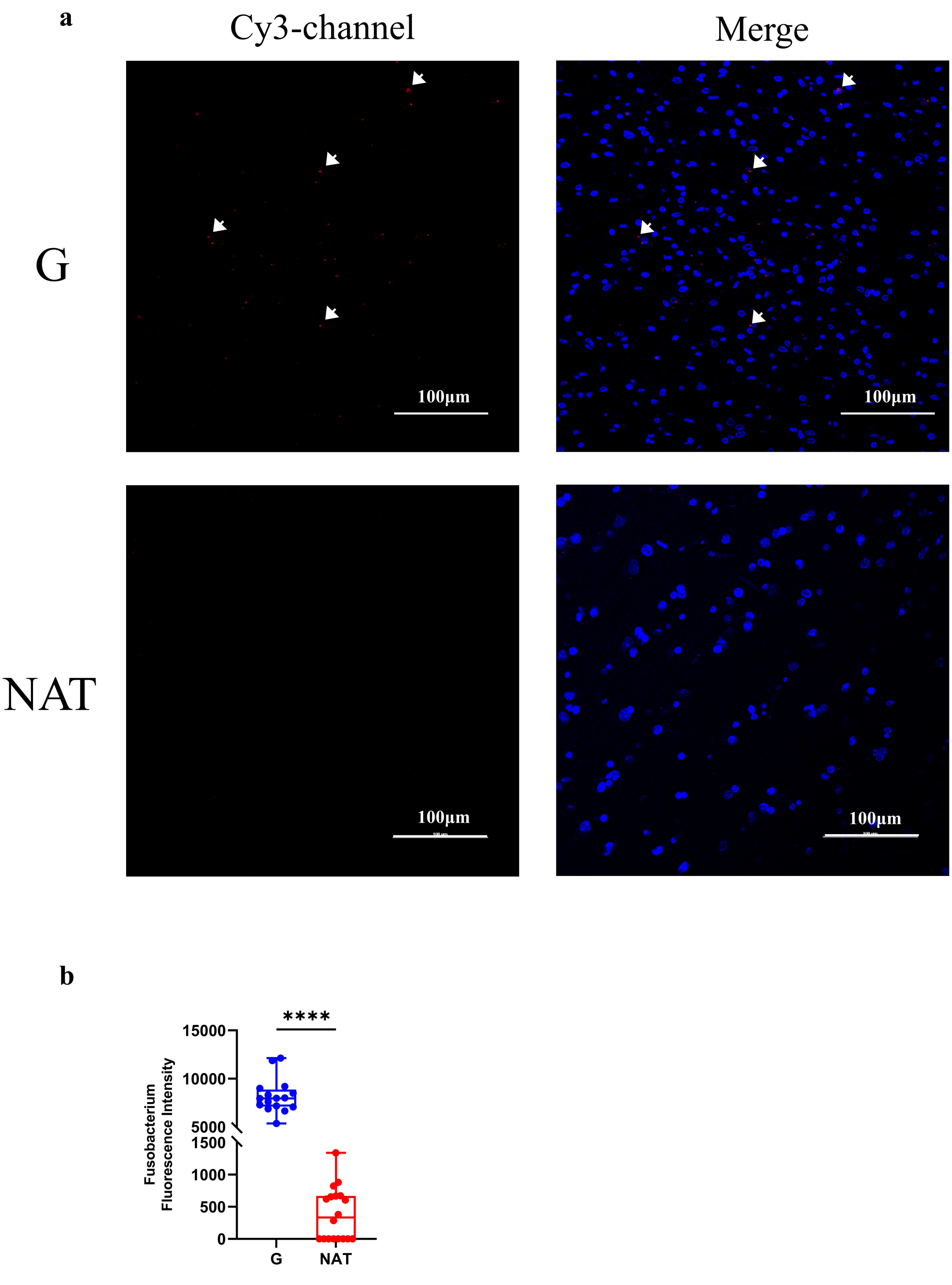

Figure S8 FISH staining of *Fusobacterium* in human glioma tissue and adjacent normal brain tissue.

a FISH staining of glioma tissue and adjacent normal brain tissue with Cy3-labeled *Fusobacterium* probe at 40X, respectively. *Fusobacterium* signals (red) and DAPI

(blue). scale bar = 100 μm.

b Statistical analysis of the levels of fluorescent signals in (A). Each dot represents one sample. G n=16; NAT n=18.; ****P < 0.0001.

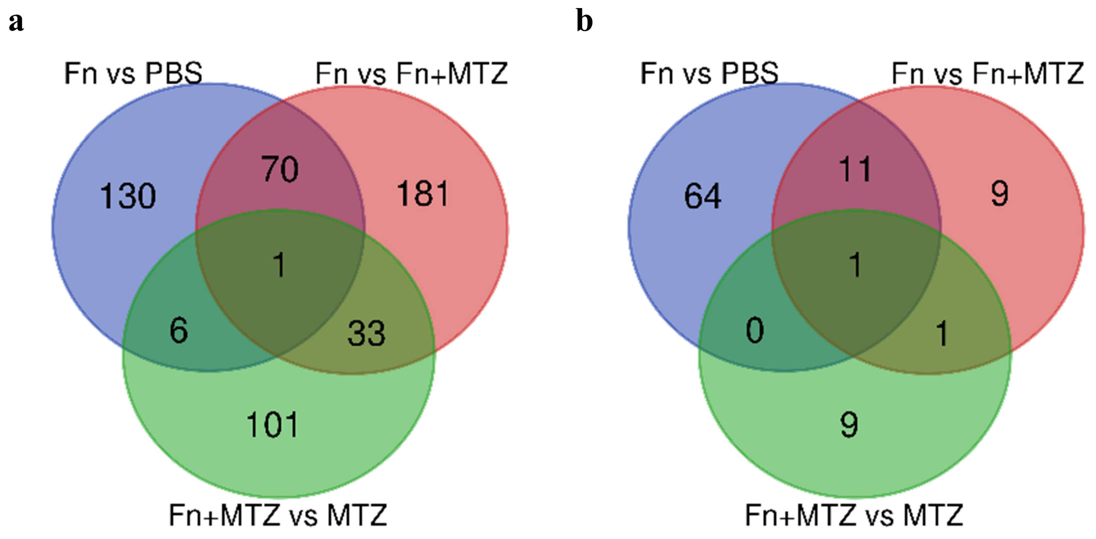

Figure S9 Analysis of differentially expressed genes and differential metabolites in mice glioma tissues.

a Summary of differentially expressed genes (determined by $P < 0.05$) between comparisons.

b Summary of differentially metabolites (determined by $P < 0.05$) between comparisons.

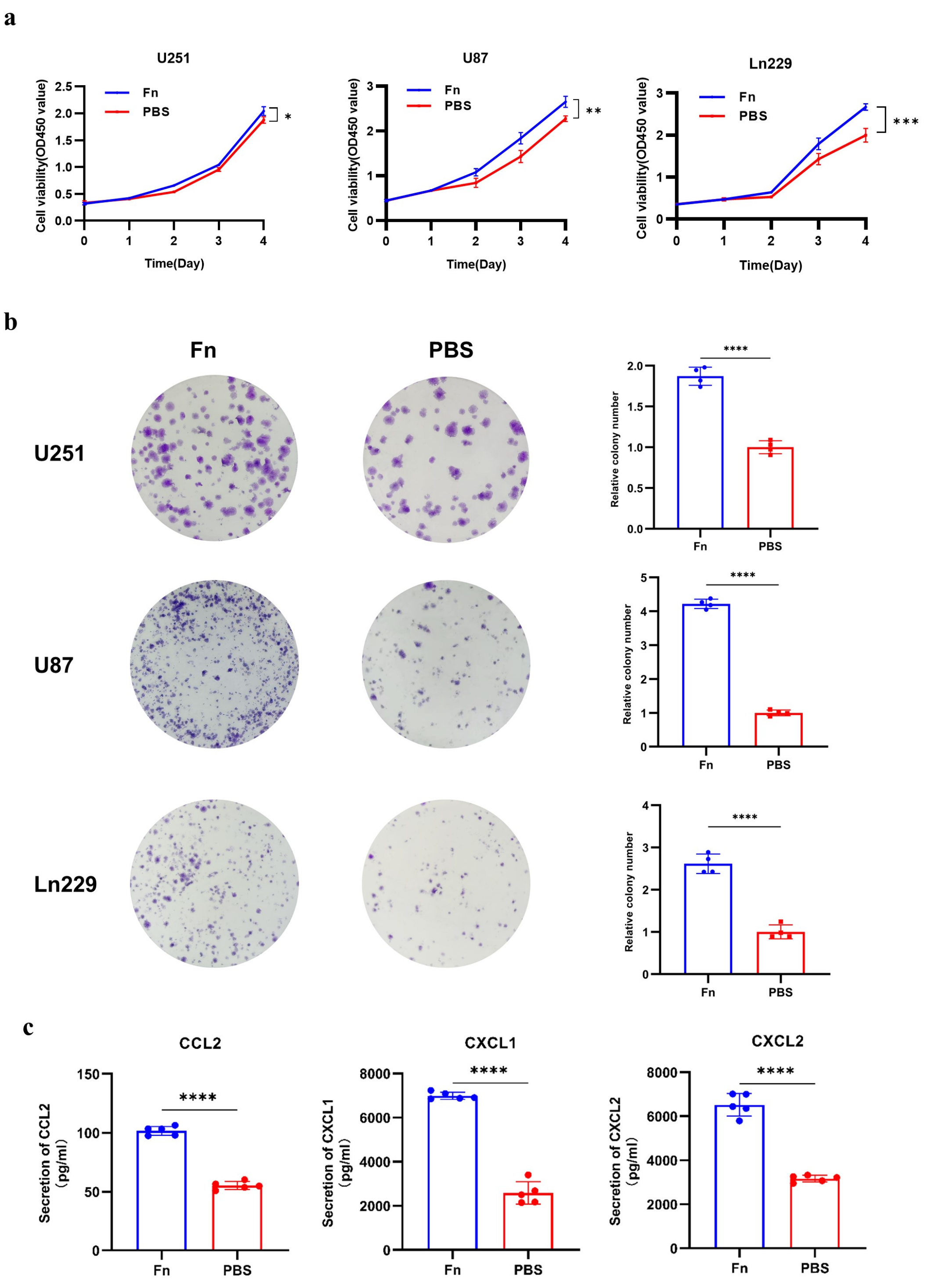

Figure S10 *Fusobacterium nucleatum* improves glioma cells proliferation.

a Proliferation of U251, U87, and Ln229 glioma cell lines in Fn and PBS groups at different time points, measured by CCK-8 assay (n=5). Statistical method: One-way

ANOVA followed by Bonferroni's multiple comparison test.

b Colony formation of U251, U87, and Ln229 glioma cell lines in Fn and PBS groups, and relative colony numbers compared at the same time point (n=4). Statistical method: Student's t-test.

c Protein expression levels of CCL2, CXCL1, and CXCL2 in the U87 glioma cell line in Fn and PBS groups, measured by ELISA (n=5). Statistical method: Student's t-test. *(P < 0.05), **(P < 0.01), ***(P < 0.001), ****(P < 0.0001), Fn, (Fusobacterium nucleatum)
