# Supplementary material for: Multi-omics analysis reveals the interplay between intratumoral bacteria and glioma
Source: mSystems. 2024 Dec 11;10(1):e00457-24. doi: 10.1128/msystems.00457-24 (PMC11748541; doi:10.1128/msystems.00457-24)
Supplement: Supplemental Methods — Additional details for multiplex immunofluorescent assay, 16S rRNA FISH, DNA extraction and sequencing, mRNA-seq, and metabolomics. [file msystems.00457-24-s0002.pdf]

Multi-omics analysis reveals interplays between intratumoral bacteria and glioma

Ting Li[1,2†], Zhanyi Zhao[1,2†], Meichang Peng[1,2], Lu Zhang[1,2], Cheng Wang[1,2], Feiyang Luo[1], Meiqin Zeng[1,2], Kaijian Sun[1,2], Zhencheng Fang[1], Yunhao Luo[1], Yugu Xie[1], Cui Lv[1], Jiaxuan Wang[1], Jian-Dong Huang[1,4,5,6,7], Hongwei Zhou[1], Haitao Sun[1,2,3]

[1]Clinical Biobank Center, Microbiome Medicine Center, Department of Laboratory Medicine, Guangdong Provincial Clinical Research Center for Laboratory Medicine, Zhujiang Hospital, Southern Medical University, Guangzhou, China

[2]Neurosurgery Center, The National Key Clinical Specialty, The Engineering Technology Research Center of Education Ministry of China on Diagnosis and Treatment of Cerebrovascular Disease, Guangdong Provincial Key Laboratory on Brain Function Repair and Regeneration, The Neurosurgery Institute of Guangdong Province Zhujiang Hospital, Southern Medical University, Guangzhou, China

[3]Key Laboratory of Mental Health of the Ministry of Education, Guangdong-Hong Kong-Macao Greater Bay Area Center for Brain Science and Brain-Inspired Intelligence, Southern Medical University, Guangzhou, China

[4]School of Biomedical Sciences, Li Ka Shing Faculty of Medicine, University of Hong Kong, Hong Kong Special Administrative Region, China

[5]Chinese Academy of Sciences (CAS) Key Laboratory of Quantitative Engineering Biology, Shenzhen Institute of Synthetic Biology, Shenzhen Institutes of Advanced

Technology, Chinese Academy of Sciences, Shenzhen, China

[6]Clinical Oncology Center, Shenzhen Key Laboratory for cancer metastasis and personalized therapy, The University of Hong Kong-Shenzhen Hospital, Shenzhen, China

[7]Guangdong-Hong Kong Joint Laboratory for RNA Medicine, Sun Yat-Sen University, Guangzhou, China

[†]These authors contributed equally to this work.

Corresponding Author:

Haitao Sun, Neurosurgery Center, Department of Laboratory Medicine, Clinical Biobank Center, Microbiome Medicine Center, Zhujiang Hospital, Southern Medical University, Guangzhou 510282, China

Email: 2009sht@smu.edu.cn

Journal name:

mSystems

Supplementary Methods

Multiplex Immunofluorescent Assay

Endogenous peroxidase was quenched with 3% H_2O_2 for 30 minutes, and then blocked with blocking reagent at room temperature for 30 minutes. Primary antibody was incubated overnight in a humidified chamber at 37 °C, washed with PBS, 3x15min, and then used HRP-conjugated secondary antibody (1:500), incubated at room temperature for 30min, washed with PBS, 3x15min, and then stained with TSA-bifluorescein (no more than 60s). Then, the slides were placed in recovery/wash buffer (Abcracker) and boiled at high power in a microwave oven until the repair solution boiled, maintained for 10 seconds and turned off, and the beaker could be placed in a water bath in a basin after 5 minutes to cool to room temperature. In order, each antigen was labeled with a different fluorescent group.

16S rRNA FISH

Slides were washed with 1xPBS (2x10 min, at RT) and then treated with HCl (0.2 N, 20 min) and Proteinase K (50μg/mL, 20 min) at RT. Slides were washed with 1xPBS (1x5 min, at RT) and then incubated with 200μL blocking buffer for 2 h at 55 °C. Slides were washed in PBS for 5 min and air-dried. The probe solutions (1:100 dilution, 250nM) were prepared by mixing probes with 25% hybridization buffer and hybridized for 72 h at 37 °C. Then, slides were washed in pre-warmed washing buffer (60°C) for 15 min and air-dried for 20 min. Finally, slides were mounted with 20 μL DAPI-Antifade solution for 10 min and covered with a cover slide in dark.

DNA extraction and sequencing

PCR amplification of the bacterial 16S rRNA gene V3-V4 regions was performed using the forward primer 341F (5'–CCTAYGGGRBGCASCAG–3') and the reverse primer 806R (5'–GGACTACNNGGGTATCTAAT–3'). Sample-specific 6-bp barcodes were incorporated into the primers for multiplex sequencing. All PCR reactions were carried out with 15 μL of Phusion® High-Fidelity PCR Master Mix (New England Biolabs), 0.2 μM of forward and reverse primers, and about 10 ng template DNA. Thermal cycling consisted of initial denaturation at 98 °C for 1 minute, followed by 30 cycles of denaturation at 98 °C for 10s, annealing at 50 °C for 30s, elongation at 72 °C for 30 s, and finally 72 °C for 5 min. PCR amplicons were purified with Qiagen Gel Extraction Kit (Qiagen, Germany) by agarose gel electrophoresis (2%). Sequencing libraries were generated using TruSeq® DNA PCR-Free Sample Preparation Kit (Illumina, USA) following the manufacturer's recommendations. The library quality was assessed on the Qubit@2.0 Fluorometer (Thermo Scientific) and Agilent Bioanalyzer 2100 system. The paired-end sequencing (2x250 bp) was performed on the Illumina NovaSeq platform (Novogene, Tianjin). Pairing and reading samples according to their unique bar codes, and cutting off the bar codes and primer sequences. We used FLASH software to merge partner readings. Quality filtering is carried out on the original reading under specific filtering conditions, and high-quality clean labels are obtained according to the formula (v0.94). EasyAmplicon (v1.12) was used to analyze downstream Amplicon bioinformatics. The full-length dereplication (derep_fulllength) command used VSEARCH (v2.15.2). Then, the Unoise3 command of USEARCH (v10.0.240) was

used to denoise the non-redundant sequences into Amplicon sequence variation (ASVs). VSEARCH (v2.15.2) was also used for reference-based chimera detection. A representative sequence was selected for each ASV, and the SILVA reference database was used to annotate taxonomic information.

mRNA-seq

The RNA sequencing was performed on the Illumina Nova6000 sequencer (Illumina). Sequencing data were dynamically removed and splice sequence fragments and low-quality fragments from the 3' end using Skewer software (v0.2.2), and preprocessed data were analyzed for quality control as well as counting of base ratios of Q20 and Q30 using FastQC software (v0.11.5). For each sample, sequence comparison of the preprocessed sequences with the reference genome sequence of the sequenced species was performed using STAR software (2.5.3a). RSEQC (v2.6.4) was used to count the comparisons. Transcripts were assembled from the reads comparison to the reference genome using StringTie software (v1.3.1c), and the new transcripts obtained from the assembly were classified. The Transdecoder program (3.0.1) was used to predict potential protein coding regions of transcripts based on Markov model principles. The expression levels for each gene were normalized to fragments per kilobase mapped （FPKM） to compare mRNA abundance between samples.

Metabolomics

25 mg of sample was weighted to an EP tube, and 500 μL extract solution (methanol: acetonitrile: water = 2: 2: 1, with isotopically-labelled internal standard mixture) was

added. Then the samples were homogenized at 35 Hz for 4 min and sonicated for 5 min in ice-water bath. The homogenization and sonication cycle were repeated for 3 times. Then the samples were incubated for 1 h at -40 °C and centrifuged at 12000 rpm for 15 min at 4 °C. The resulting supernatant was transferred to a fresh glass vial for analysis. LC-MS/MS analyses were performed using an UHPLC system (Vanquish, Thermo Fisher Scientific) with a UPLC BEH Amide column (2.1 mm × 100 mm, 1.7 μm) coupled to Orbitrap Exploris 120 mass spectrometer (Orbitrap MS, Thermo). The mobile phase consisted of 25 mmol/L ammonium acetate and 25 mmol/L ammonium hydroxide in water（pH = 9.75）(A) and acetonitrile (B). The auto-sampler temperature was 4 °C, and the injection volume was 2 μL. The Orbitrap Exploris 120 mass spectrometer was used for its ability to acquire MS/MS spectra on information-dependent acquisition (IDA) mode in the control of the acquisition software (Xcalibur, Thermo). The raw data were converted to the mzXML format using ProteoWizard and processed with an in-house program, which was developed using R and based on XCMS, for peak detection, extraction, alignment, and integration. Then an in-house MS2 database (Biotree Biomedical Technology Corporation, Shanghai, China) was applied in metabolite annotation. The cutoff for annotation was set at 0.3.

In this experiment, SIMCA software (v16.0.2) was used to perform logarithmic transformation plus centering formatting on the data, and then automatic modeling analysis was performed to obtain the principal component analysis (PCA) model. Next, the data were log-transformed plus UV formatted using SIMCA software (v16.0.2), and the first principal component was first analyzed by OPLS-DA modeling.
